# The *Airn* lncRNA does not require any DNA elements within its locus to silence distant imprinted genes

**DOI:** 10.1371/journal.pgen.1008268

**Published:** 2019-07-22

**Authors:** Daniel Andergassen, Markus Muckenhuber, Philipp C. Bammer, Tomasz M. Kulinski, Hans-Christian Theussl, Takahiko Shimizu, Josef M. Penninger, Florian M. Pauler, Quanah J. Hudson

**Affiliations:** 1 CeMM, Research Center for Molecular Medicine of the Austrian Academy of Sciences, Vienna, Austria; 2 IMP/IMBA Transgenic Service, Institute of Molecular Pathology (IMP), Vienna, Austria; 3 National Center for Geriatrics and Gerontology, Obu Aichi, Japan; 4 IMBA, Institute of Molecular Biotechnology of the Austrian Academy of Sciences, Vienna, Austria; 5 Department of Obstetrics and Gynecology, Medical University of Vienna, Vienna, Austria; University of Pennsylvania, UNITED STATES

## Abstract

Long non-coding (lnc) RNAs are numerous and found throughout the mammalian genome, and many are thought to be involved in the regulation of gene expression. However, the majority remain relatively uncharacterised and of uncertain function making the use of model systems to uncover their mode of action valuable. Imprinted lncRNAs target and recruit epigenetic silencing factors to a cluster of imprinted genes on the same chromosome, making them one of the best characterized lncRNAs for silencing distant genes *in cis*. In this study we examined silencing of the distant imprinted gene *Slc22a3* by the lncRNA *Airn* in the *Igf2r* imprinted cluster in mouse. Previously we proposed that imprinted lncRNAs may silence distant imprinted genes by disrupting promoter-enhancer interactions by being transcribed through the enhancer, which we called the enhancer interference hypothesis. Here we tested this hypothesis by first using allele-specific chromosome conformation capture (3C) to detect interactions between the *Slc22a3* promoter and the locus of the *Airn* lncRNA that silences it on the paternal chromosome. In agreement with the model, we found interactions enriched on the maternal allele across the entire *Airn* gene consistent with multiple enhancer-promoter interactions. Therefore, to test the enhancer interference hypothesis we devised an approach to delete the entire *Airn* gene. However, the deletion showed that there are no essential enhancers for *Slc22a2*, *Pde10a* and *Slc22a3* within the *Airn* gene, strongly indicating that the *Airn* RNA rather than its transcription is responsible for silencing distant imprinted genes. Furthermore, we found that silent imprinted genes were covered with large blocks of H3K27me3 on the repressed paternal allele. Therefore we propose an alternative hypothesis whereby the chromosome interactions may initially guide the lncRNA to target imprinted promoters and recruit repressive chromatin, and that these interactions are lost once silencing is established.

## Author summary

Long non-coding (lnc) RNAs are numerous in the mammalian genome and many have been implicated in gene regulation. However, the vast majority are uncharacterised and of uncertain function making known functional lncRNAs valuable models for understanding their mechanism of action. One mode of lncRNA action is to recruit epigenetic silencing to target distant genes on the same chromosome. A well-characterized group of lncRNAs that act in this way to silence genes are imprinted lncRNAs. In this study we examined how the imprinted lncRNA *Airn* silences genes in the *Igf2r* imprinted cluster, focusing primarily on silencing of the distant imprinted gene *Slc22a3*. We found that *Airn* expression blocks chromosome interactions between the *Slc22a3* promoter and the *Airn* gene locus. By making a large genomic deletion including the *Airn* gene we showed that these interactions are not essential enhancer/promoter interactions, but may help to guide the *Airn* RNA to target genes to recruit epigenetic silencing. Our study adds to the understanding of how lncRNAs may act to silence distant genes.

## Introduction

Long non-coding (lnc) RNAs are a diverse and numerous group of non-protein-coding RNA species longer than 200 nucleotides, some of which have been shown to be involved in gene regulation [1,2]. A growing number of lncRNAs have been implicated in development and disease, sparking interest in how they may regulate gene expression [3,4]. However, the majority of lncRNAs remain relatively uncharacterized and of uncertain function, highlighting the value of model systems to identify modes of lncRNA action. One of the most studied functional lncRNAs in mammals are imprinted lncRNAs, which are expressed exclusively from either the maternally or paternally inherited chromosome. Mechanisms of lncRNA action identified in imprinted lncRNAs, such as the targeting of histone modifying complexes to genomic loci and the role of lncRNA transcription in gene regulation [5–8], have later been shown for other non-imprinted lncRNAs [2,9], emphasizing their value as model systems.

Genomic imprinting is an epigenetic mechanism that restricts gene expression to one of the two parental alleles. Imprinted genes are often clustered in domains with a differentially DNA methylated genetic region called the imprint control element (ICE, also called the imprinting control region (ICR)) controlling allele specific expression of all genes in the cluster [10]. Although differential methylation of the ICE is established during gametogenesis and maintained through somatic cell division, the extent of imprinted silencing is dynamic throughout development, with imprinted clusters tending to show their maximum size in extra-embryonic tissues like the visceral yolk sac (VYS) and the placenta [11]. For example, the *Igf2r* cluster expands from 120kb in most embryonic and adult tissues to almost 10Mb in placenta, while the *Kcnq1* cluster expands from 250kb in embryonic tissues to 690kb in VYS [11]. The number of imprinted genes in mammals appears to be limited to approximately one hundred [11], a number of which have been shown to be key regulators of development and disease [12,13].

Mechanistically ICEs often act as promoters for a lncRNA, with the imprinted lncRNA being expressed from the non-methylated allele initiating silencing *in cis* of all genes in the cluster [14]. This has been shown in mouse by truncating the lncRNA to a non-functional length for *Airn*, *Kcnq1ot1*, *Nespas* and *Ube3a-ATS* in the *Igf2r*, *Kcnq1*, *Gnas* clusters and the orthologous cluster to the human Prader-Willi/Angleman region respectively [5,6,15,16] One of the best-characterized clusters is the *Igf2r* cluster where the *Airn* lncRNA causes imprinted silencing of *Igf2r* in most tissues, and a larger cluster of genes in extra-embryonic tissues [5,11,17]. The function of the *Airn* lncRNA was previously tested using two mouse models that ablate imprinted silencing in the *Igf2r* cluster: a deletion of the *Airn* promoter and ICE (R2Δ), and the truncation of *Airn* by the insertion of a polyadenylation signal (*AirnT)* [5,18]. Using the *AirnT* model we showed that imprinted silencing in VYS extends over 450kb to *Slc22a2* and *Slc22a3* [17], while more recently we used the R2Δ model to show that in placenta the domain of genes showing imprinted silencing by *Airn* extends over 10Mb, making it the largest imprinted cluster known [11].

*Airn* overlaps *Igf2r* in antisense and silences it by transcriptional interference [7], but how non-overlapped imprinted genes in the cluster are silenced is disputed. In trophoblast stem (TS) cells the silenced paternal *Igf2r* cluster expressing *Airn* is contracted and associated with a so-called repressive domain that includes the polycomb repressive complex (PRC) modifications H3K27me3 (PRC2) and H2AK119u1 (PRC1) together with the PRC1 protein Rnf2 [19]. There is also some evidence that *Airn* may bind PRC2 [20]. In placenta *Airn* binds the H3K9 dimethylase EHMT2 (also known as G9a), which is enriched on the *Slc22a3* promoter, and required for *Slc22a3* imprinted silencing [21]. The *Airn* RNA is closely associated with the *Slc22a3* promoter in placenta, indicating that *Airn* may target EHMT2 to the *Slc22a3* promoter to cause silencing [21]. These data indicate that the *Airn* RNA product may silence non-overlapped imprinted genes like *Slc22a3* by targeting EHMT2 and perhaps PRC2/PRC1 to their promoters. However, given that this contrasts with the mechanism of *Igf2r* silencing, where *Airn* transcription and not its RNA product mediate silencing, we have proposed an alternative hypothesis to explain these data. Enhancers form specific chromosome interactions with promoters to activate them [22], therefore we hypothesized that *Airn* transcription may prevent upregulation of non-overlapped imprinted genes like *Slc22a3* by interfering with enhancer access to their promoters, and that as a secondary step EHMT2 and PRC2/PRC1 may deposit repressive chromatin modifications to maintain silencing [23]. Consistent with this we found enrichment of the active enhancer marker H3K27ac within the *Airn* gene in VYS endoderm and placenta, and open chromatin within the *Airn* gene in multiple tissues [11,24]. Enhancers often lie in the introns of actively transcribed genes and are not disturbed by transcription through them. We hypothesize that the RNA polymerase transcribing *Airn* has unique properties, as *Airn* lncRNA has unusual RNA biology features like a lack of splicing, nuclear retention and a short half-life [25,26]. It is therefore possible that this specific RNA polymerase complex enables not only transcriptional interference with the *Igf2r* promoter, but also transcriptional interference with enhancers [7,23].

In this paper we aimed to test the enhancer interference hypothesis. First we determined if the predicted chromosome interactions could be detected, and second we performed a genetic test to determine if disrupting the predicted enhancers affected expression of imprinted genes in the *Igf2r* cluster.

## Results

### *Airn* blocks chromosome interactions with the upstream imprinted gene *Slc22a3*

The enhancer interference model hypothesizes that transcription of *Airn* through enhancers for non-overlapped imprinted genes may disrupt enhancer activity preventing upregulation of these genes [23]. This predicts that regions within the *Airn* gene should interact with non-overlapped imprinted genes on the active maternal allele, and not on the paternal allele where expression of *Airn* causes imprinted silencing. To test this we conducted chromosome conformation capture (3C) to compare interactions on the maternal and paternal alleles between the promoter of the non-overlapped imprinted gene *Slc22a3*, lying 234 kb upstream of *Airn*, and the *Airn* gene body. We chose to examine *Slc22a3* because it is the only non-overlapped imprinted gene in the *Igf2r* cluster to show imprinted expression in multiple tissue types [11], and in order to compare to other studies where regulation of *Slc22a3* imprinted expression was examined [19,21].

**Fig 1:**
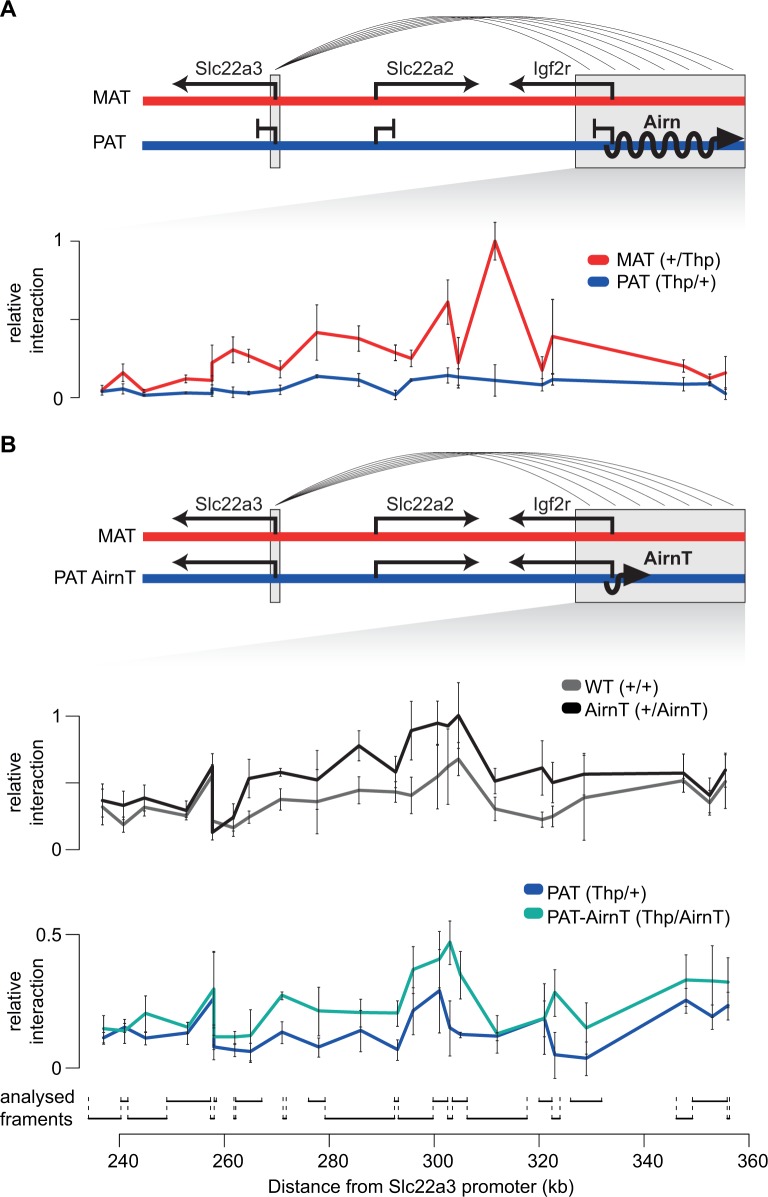
Chromosome Conformation Capture (3C) indicates that the *Airn* gene body may contain multiple enhancers for *Slc22a3*. (A) Chromosome interactions between the *Slc22a3* promoter and the *Airn* gene body are enriched on the maternal allele. Top: In the *Igf2r* imprinted cluster in visceral yolk sac (VYS), *Slc22a3*, *Slc22a2* and *Igf2r* are expressed from the maternal (red) and repressed on the paternal (blue) allele. Long arrows indicate active expression, blocked arrows indicate repression. The *Slc22a3* promoter region (3C bait fragment) and *Airn* gene body (3C prey fragments) are indicated by grey boxes. Multiple lines indicate the interactions assayed by 3C. Bottom: The relative level of 3C interactions identified on the maternal (red, +/*Thp*) and paternal (blue, *Thp*/+) alleles. (B) Paternal allele chromosome interactions between the *Slc22a3* promoter and the *Airn* gene body are increased following truncation of *Airn*. Top: Imprinted silencing in the *Igf2r* cluster in the VYS is lost following truncation of *Airn* (*AirnT*, colors and arrows as in A). Middle: The relative level of 3C interactions identified in the wildtype (black, +/+) and *AirnT* (grey, +/*AirnT*) mice (both parental alleles present). Bottom: The relative level of 3C interactions detected on the wildtype (dark blue, *Thp*/+) and the *AirnT* (cyan, *Thp*/*AirnT*) paternal alleles. 3C interactions were determined using Taqman qPCR, normalized to the mean of 2 interactions in the *Igf2* cluster, and then the highest interaction for A and B were set to 1, as detailed in the methods. Positions and size of prey fragments investigated in the 3C assay are indicated at the bottom. Points and error bars are mean and standard deviation of 3 technical replicates.

*Slc22a3* shows imprinted expression in both the placenta and VYS, but we chose to use VYS for the 3C analysis, as it is a simpler tissue that contains no maternal cells [17]. To enable parental allele specific analysis, we collected VYS from reciprocal crosses of the spontaneous T-hairpin mutant mouse (*Thp*) [27], which has a large deletion (minimum 5.56 Mb) that includes the *Igf2r* cluster [28]. We found that interactions between the *Slc22a3* promoter and *Airn* are higher on the maternal allele across the entire *Airn* gene (Fig 1A). This indicates that *Airn* blocks these interactions on the paternal allele consistent with the enhancer interference model.

To test this, we conducted a second 3C experiment to determine if loss of *Airn* would restore interactions between *Slc22a3* and the *Airn* gene on the paternal allele. We collected VYS from a cross between *Thp* mice and mice with a truncation of *Airn* (*AirnT*) that leads to a loss of imprinted silencing [5]. This enabled us to compare interactions between the *Slc22a3* promoter and *Airn* gene in the presence and absence of a functional *Airn*. We found that truncation of *Airn* led to an increase in interactions with the whole *Airn* gene, both for the biallelic comparison (+/+ vs +/*AirnT*) and for the comparison where only the paternal allele was present (*Thp*/+ vs *Thp*/*AirnT*) (Fig 1B). This indicates that *Airn* interferes with interactions between its gene body and the *Slc22a3* promoter, as predicted by the enhancer interference model.

### Large genomic deletion indicates that *Airn* contains no essential enhancers for *Slc22a3*

**Fig 2:**
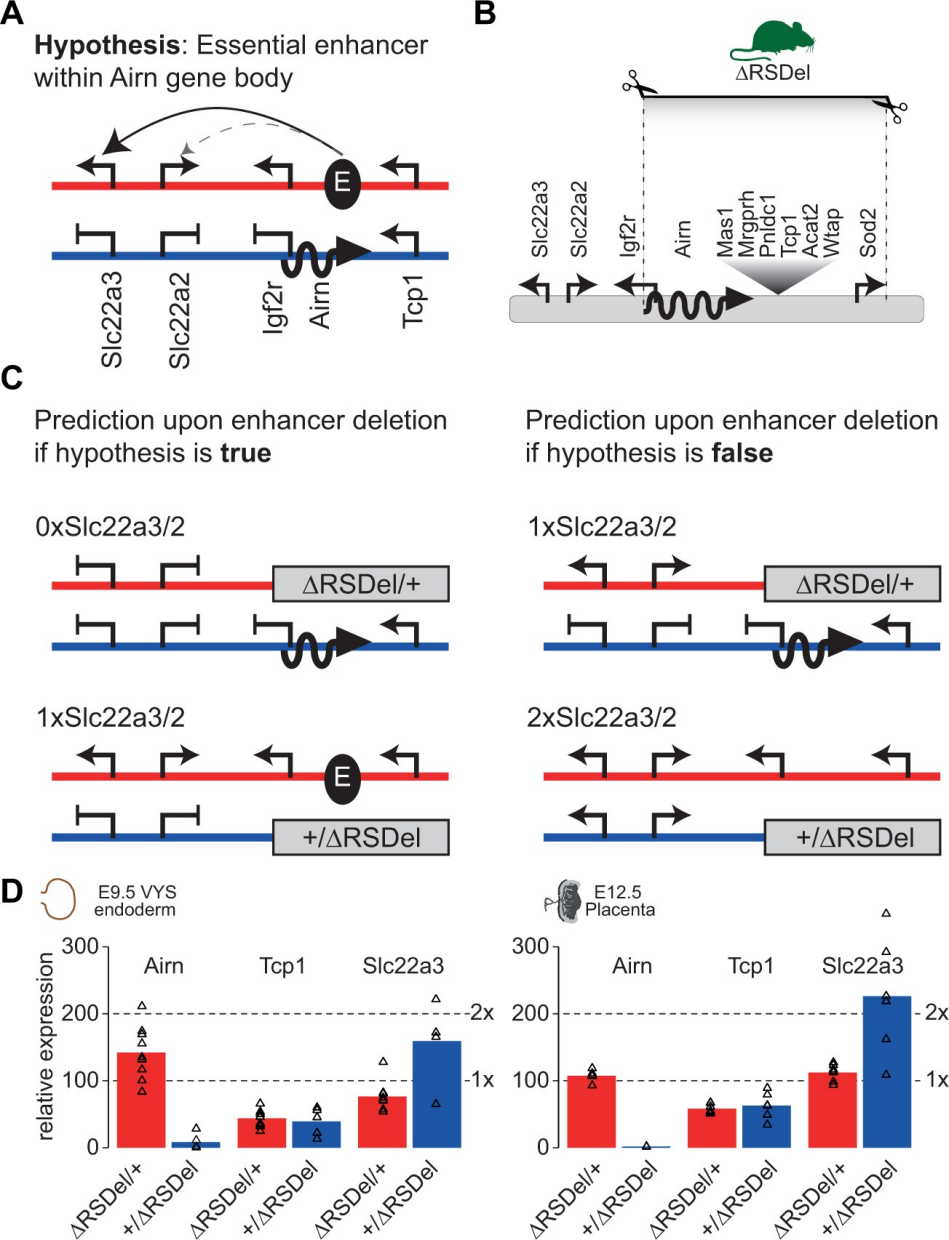
Deletion of the *Airn* gene indicates that it contains no essential enhancers for *Slc22a3*. (A) The enhancer interference hypothesis. The *Airn* gene body contains an essential enhancer (E) that interacts with the *Slc22a3* promoter (solid arrow) and potentially also with the *Slc22a2* promoter (dashed arrow) on the maternal allele (red) activating gene expression (arrows). On the paternal allele (blue) *Airn* (wavy line) prevents this interaction causing silencing (blocked arrows). (B) The *RSDel* deletion spans 270 kb from the *Airn* promoter to the third intron of the *Sod2* gene, and includes the entire 118 kb *Airn* gene and 6 addition genes. The deletion was constructed by Cre-mediated *trans* recombination between loxP sites in the R2Δ *Airn* promoter deletion and the *Sod2Δ* alleles as detailed in the text. (C) Predicted expression patterns in the *RSDel* deletion that includes the entire *Airn* gene. Left: Prediction if enhancer interference hypothesis is correct: The maternal deletion removes the essential enhancer for *Slc22a3* and *Slc22a2* preventing their upregulation, and leading to a loss of expression. The paternal deletion removes *Airn*, but also the essential enhancer, so *Slc22a3* and *Slc22a2* remain silenced on the paternal allele, leading to normal levels of expression. Right: Prediction if enhancer interference hypothesis is false: The maternal deletion has no effect on expression of *Slc22a3* and *Slc22a2*, leading to normal levels of expression. The paternal deletion removes *Airn* leading to a loss of imprinted silencing on the paternal allele, and a doubling of *Slc22a3* and *Slc22a2* expression. (D) The *RSDel* maternal deletion does not affect *Slc22a3* expression, whereas the paternal deletion leads to a doubling of *Slc22a3* expression. RT-qPCR expression analysis of the *RSDel* maternal deletion (red, *RSDel*/+) and paternal deletion (blue, +/*RSDel*) in E9.5 VYS endoderm (left) and E12.5 placenta (right). Expression levels are normalized to wildtype for each cross (set to 100). Bars show the mean and triangles indicate all data points (biological replicates). Note that *Airn* and *Tcp1* (non-imprinted gene) are within the deletion while *Slc22a3* is outside.

The enhancer interference model predicts that essential enhancers for *Slc22a3* should lie within the *Airn* gene, and that transcription of *Airn* through these enhancers should prevent upregulation of *Slc22a3* on the paternal allele (Fig 2A). This is supported by an enrichment in maternal interactions between the *Slc22a3* promoter and the *Airn* gene in VYS (Fig 1), along with a broad enrichment of the active enhancer mark H3K27ac across the *Airn* gene in VYS endoderm and placenta [11], and multiple regions of open chromatin within the *Airn* gene other tissues [24]. Therefore, to test the enhancer interference model we devised an approach to delete the entire *Airn* gene in a mouse.

We chose to take advantage of existing mouse strains to engineer a deletion of *Airn* by targeted recombination during male meiosis [29]. We bred together the *Airn* promoter deletion mouse (*R2Δ*) with a *Sod2* exon 3 deletion mouse (*Sod2Δ*) and the Hprt-Cre mouse [18,30,31]. Both *R2Δ* and *Sod2Δ* contained a single loxP site in the same orientation, which enables Cre mediated *trans* recombination during male meiosis to generate either a deletion or duplication of the 270kb intervening region, including the entire 118kb *Airn* gene (Fig 2B) [29]. By mating males containing all 3 alleles with wildtype females, and screening 72 offspring we were able to identify 1 male founder that contained the deletion, which we then used to establish the *RSDel* strain (*R2Δ* to *Sod2Δ* deletion).

If the hypothesis that essential enhancers are present within the *RSDel* region is correct, deletion of these enhancers on the maternal allele where *Airn* is not expressed should prevent expression of *Slc22a3* on this chromosome. *Slc22a3* silencing should also be maintained on the paternal allele when these enhancers are deleted (Fig 2C left). This would be in contrast to all other mutations of the *Airn* gene that disrupt *Airn* expression, but do not delete potential enhancers, and that lead to a loss of imprinted silencing [5,7,18,32]. Alternatively, if the hypothesis is false, we would expect that deletion of candidate regions on the maternal allele would not affect *Slc22a3* expression, whereas deletion of the paternal allele would lead to a loss of imprinted silencing, similar to other *Airn* mutants (Fig 2C right).

To assess the effect of the *RSDel* deletion on *Slc22a3* expression we collected embryonic tissue from reciprocal crosses to wildtype FVB mice. We isolated the VYS endoderm layer to focus on the most relevant cell type where *Slc22a3* shows imprinted expression [17]. We found that when the deletion was maternally inherited there was no effect on *Slc22a3* expression, whereas when the deletion was paternally inherited *Slc22a3* expression doubled (Fig 2D left). This correlated with a loss of *Airn* expression, indicating this increase in expression was due to a loss of imprinted expression, as with other *Airn* mutants [5,7,18,32]. The non-imprinted gene *Tcp1* that lies within the *RSDel* deletion showed a similar expression level whether the deletion was inherited maternally or paternally (Fig 2D left). Similarly, in placenta where *Slc22a3* also shows imprinted expression, the maternal deletion did not affect *Slc22a3* imprinted expression, but the paternal deletion and loss of *Airn* expression led to a doubling of *Slc22a3* expression, whereas *Tcp1* showed a similar level of expression in both deletions (Fig 2D right).

**Fig 3:**
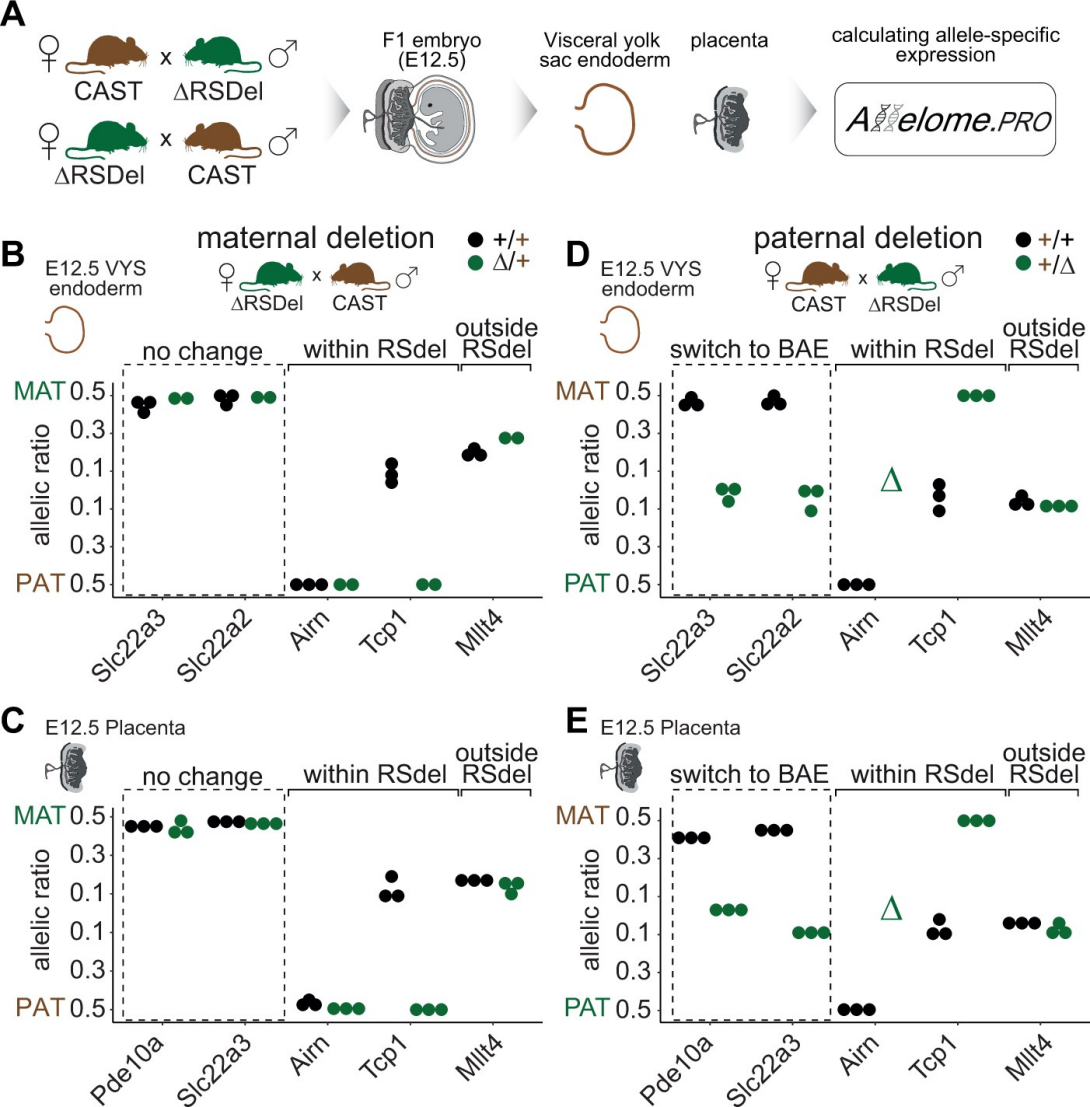
Allele-specific expression analysis shows imprinted expression is unaffected by maternal deletion of the *Airn* gene, but lost upon paternal deletion. (A) VYS endoderm and placenta was isolated from E12.5 F1 embryos from *RSDel* x CAST reciprocal crosses and subject to RNA-seq (3 wildtype and 3 *RSDel* from each cross and tissue). The data was subject to allelic expression analysis using the Allelome.PRO pipeline [33] as detailed in the methods. (B) The maternal *RSDel* deletion does not affect imprinted expression of *Slc22a3* and *Slc22a2* in VYS endoderm. Within the deletion *Airn* is unaffected as it is exclusively paternally expressed, while the non-imprinted gene *Tcp1* becomes paternally expressed, and *Mllt4* lying 750kb outside of the deletion is unaffected. (C) The maternal *RSDel* deletion does not affect imprinted expression of *Slc22a3* and *Pde10a* in placenta. Within the deletion *Airn* and *Tcp1*, show paternal expression, while *Mllt4* is unaffected. (D) The paternal *RSDel* deletion leads to loss of *Slc22a3* and *Slc22a2* imprinted expression in VYS endoderm. Within the deletion *Airn* expression is lost, as it is shows exclusive paternal expression, while *Tcp1* shows maternal expression, and *Mllt4* outside the deletion is unaffected. (E) The paternal *RSDel* deletion leads to loss of *Slc22a3* and *Pde10a* imprinted expression in placenta. *Airn* expression is lost, *Tcp1* becomes maternally expressed, and Mllt4 is unaffected.

To directly test the effect of the *RSDel* deletion on imprinted expression, and to extend our analysis to other genes in the *Igf2r* imprinted cluster, we performed allele-specific expression analysis on RNA-seq of embryonic tissue collected from reciprocal crosses between *RSDel* and the genetically distinct CAST mice (Fig 3A). We used the Allelome.PRO pipeline that we previously developed to analyze expression over SNPs between these strains [11,33]. For the maternal deletion, in VYS endoderm we found that *Slc22a3*, and also *Slc22a2*, maintained maternal imprinted expression, while *Airn* within the deletion maintained paternal imprinted expression as expected. The non-imprinted *Tcp1* gene within the deletion switched to paternal expression due to loss of the maternal copy, whereas the non-imprinted *Mllt4* gene lying 750kb outside of the deletion was unaffected (Fig 3B). Similarly in placenta *Slc22a3*, *Pde10a* and *Airn* maintained imprinted expression, while *Tcp1* showed paternal only expression and *Mllt4* biallelic expression as in VYS endoderm (Fig 3C). For the paternal deletion, in VYS endoderm we observed a loss of imprinted expression for *Slc22a3* and *Slc22a2*, while *Airn* expression was completely lost as it is expressed exclusively from the paternal allele. As expected, *Tcp1* within the deletion showed maternal only expression and *Mllt4* expression was unaffected by the deletion (Fig 3D). The results in placenta were similar, with *Slc22a3* and *Pde10a* showing a loss of imprinted expression, *Airn* expression being completely lost, and *Tcp1* and *Mllt4* showing the expected expression pattern (Fig 3E).

In summary, the maternal *RSDel* deletion did not affect imprinted expression of *Slc22a2*, *Slc22a3*, and *Pde10a* in VYS endoderm and placenta (Fig 2D, Fig 3B and 3C), whereas the paternal deletion led to a loss of imprinted expression (Fig 2D, Fig 3D and 3E). These results support the alternative hypothesis (Fig 2C right), and indicate that there are no essential enhancers for *Slc22a3* expression within the *Airn* gene or the downstream region to *Sod2*.

### Broad paternal allele enrichment of H3K27me3 on imprinted genes in the *Igf2r* cluster in visceral yolk sac endoderm

**Fig 4:**
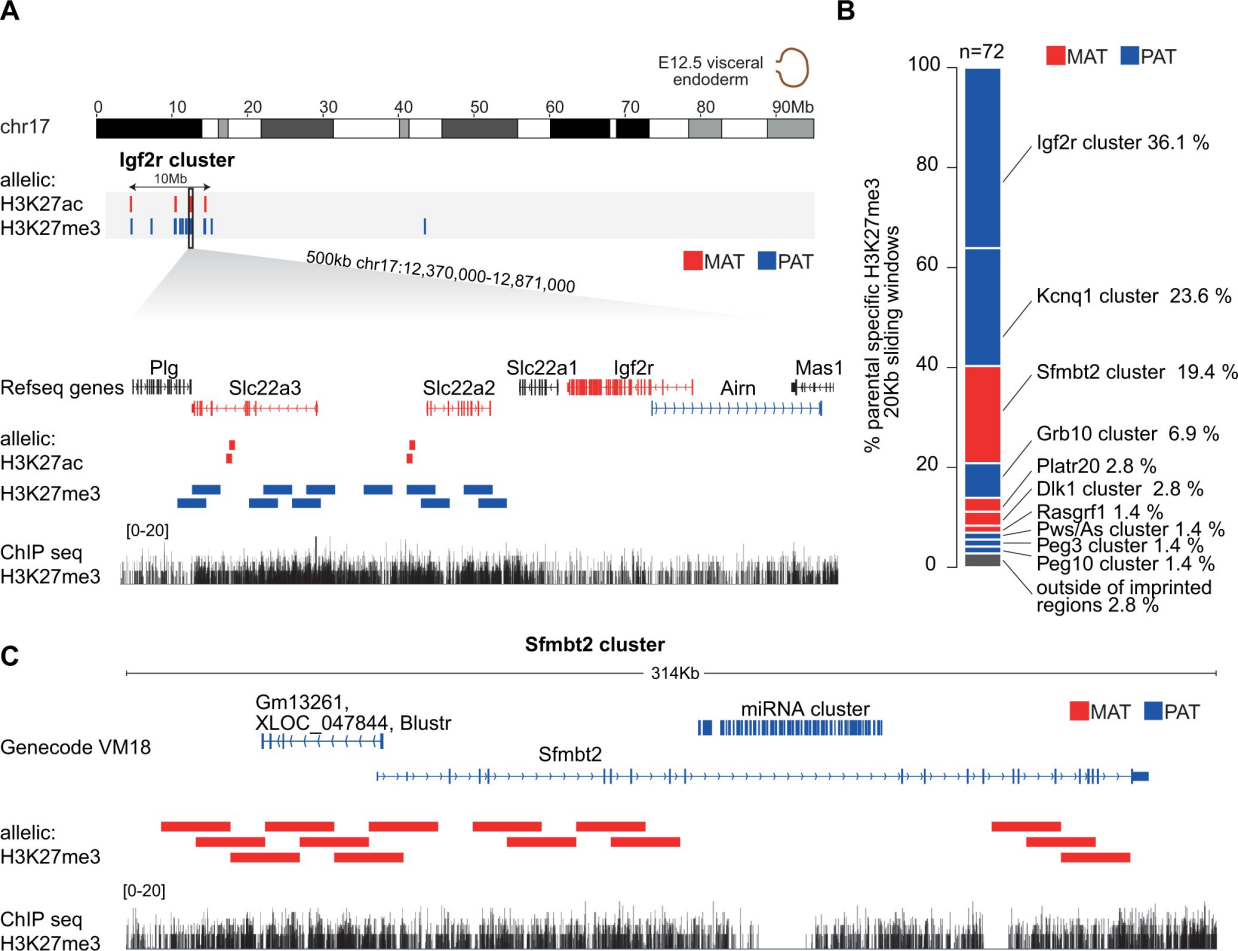
Broad enrichments of H3K27me3 cover the silenced allele of imprinted genes in VYS endoderm. (A) Maternal enrichment of H3K27ac and paternal enrichment of H3K27me3 is present at sites across the entire 10Mb *Igf2r* cluster in VYS endoderm, despite imprinted expression being limited to the 450Kb from *Slc22a3* to *Airn* in this tissue. In this region showing imprinted expression, broad enrichment of H3K27me3 covers the silenced paternal alleles of *Slc22a3* and *Slc22a2*, while more focal maternal enrichment of H3K27ac is seen within these genes. (B) Genome-wide 97.2% of H3K27me3 parental allele enriched 20kb windows in VYS endoderm lie within imprinted clusters, with the *Igf2r* cluster showing the highest number, followed by the *Kcnq1* cluster and then the *Sfmbt2* cluster. (C) H3K27me3 is enriched over the silenced maternal allele of *Sfmbt2* and *Blustr* in the *Sfmbt2* cluster.

Given that the *RSDel* deletion disproves the enhancer interference hypothesis, we sought to further investigate the predictions of alternative models of *Airn*-mediated imprinted silencing in extra-embryonic tissues. *Airn* has been proposed to recruit and target the histone modifying complexes EHMT2 and the polycomb repressive complexes 1 and 2 (PRC1 and PRC2) to distant imprinted genes in extra-embryonic tissues [19,21]. However, the parental allele specific chromosome localization of H3K27me3 has not been investigated at the *Igf2r* cluster in extra-embryonic tissues. Therefore, we performed H3K27me3 chromatin immunoprecipitation sequencing (ChIP-seq) on VYS endoderm from FVB x CAST reciprocal crosses to determine the allele-specific distribution of this mark in the *Igf2r* cluster and throughout the genome. Using the Allelome.PRO pipeline to analyze the data [11,33], we found paternal enrichment of H3K27me3 with matching H3K27ac maternal enrichment across the entire 10Mb *Igf2r* cluster, despite imprinted expression in VYS endoderm being limited to a 450kb region from *Slc22a3* to *Airn* (Fig 4A) [11]. Within this region we found broad enrichment of H3K27me3 over the silenced paternal alleles of *Slc22a3* and *Slc22a2* (Fig 4A), similar to the broad enrichment of H3K27me3 over biallelically silenced genes that we have previously reported [34]. In genome-wide analysis we found that 97% of H3K27me3 enriched windows mapped to imprinted regions, with the *Igf2r* imprinted cluster showing the greatest number of enriched windows, followed by the *Kcnq1* cluster, which has been previously reported to show paternal allele enrichment of H3K27me3 over silenced imprinted genes in placenta (Fig 4B) [8,35]. Interestingly, the *Sfmbt2* imprinted region reported to show H3K27me3 mediated DNA methylation independent imprinted expression [36], showed the third highest level of H3K27me3 parental allele specific enrichment (Fig 4B), with maternal allele enrichment over *Sfmbt2* and the *Blustr* lncRNA shown to positively regulate its expression (Fig 4C) [37]. These results are consistent with H3K27me3 playing a role in the initiation and/ or maintenance of imprinted silencing for both the lncRNA-mediated silencing that occurs in the *Igf2r* and *Kcnq1* clusters, as well as for lncRNA independent imprinted silencing, such as occurs in the *Sfmbt2* imprinted cluster.

## Discussion

LncRNA mediated imprinted silencing of one copy of genes like *Igf2r* and *Cdkn1c* is required for development, but imprinted lncRNAs also provide a tractable model system for understanding gene regulation by lncRNAs in general [10]. In this study we used imprinted silencing of the upstream imprinted genes *Slc22a2*, *Slc22a3* and *Pde10a* by the lncRNA *Airn*, as a model for how lncRNAs may silence non-overlapped distant genes *in cis*. We found chromosome interactions on the active maternal allele between the *Airn* gene body and the *Slc22a3* promoter supporting the previously proposed enhancer interference hypothesis [23]. However, a genetic test where we deleted the entire *Airn* gene demonstrated that *Airn* contains no essential enhancers for *Slc22a3* disproving this model, and requiring the development of a new hypothesis to explain the data in this and previous studies.

Previously it has been shown in placenta using a technique derived from RNA FISH called RNA TRAP (Tagging and Recovery of Associated Proteins) that *Airn* is associated with the *Slc22a3* promoter [21]. Surprisingly in this study in VYS using 3C we found an association between the *Slc22a3* promoter and the *Airn* gene on the maternal allele, and not on the paternal allele. However, these results are not contradictory, as an association between the *Airn* RNA and the *Slc22a3* promoter on the paternal allele (detectable by TRAP) is not the same as an interaction between the *Airn* genomic locus and the *Slc22a3* promoter on the maternal allele (detectable by 3C).

Here we show in VYS endoderm that the repressed alleles of *Slc22a3* and *Slc22a3* in the *Igf2r* cluster are covered by a broad enrichment of the PRC2 mark H3K27me3, as are imprinted genes in the *Kcnq1* cluster and in other imprinted clusters. Imprinted lncRNAs including *Airn*, *Kcnq1ot1* and *Meg3* have been reported to directly interact with PRC2 and EHMT2 [8,20,21], although the *Airn*-PRC2 interaction was reported in embryonic stem (ES) cells where *Airn* is very lowly expressed and no genes in the *Igf2r* cluster show imprinted expression [11,20]. PRC1, PRC2 and EHMT2 have been shown to be required to maintain imprinted silencing of members of the *Kcnq1* cluster that show extra-embryonic specific imprinted expression [19,38], and it has also been recently reported that imprinted silencing of *Dlk1* by *Meg3* requires PRC2 [39]. *Igf2r* imprinted silencing by *Airn* does not require PRC2 or EHMT2 [21,40], but while the effect of loss of PRC1 and PRC2 on *Slc22a3* and other members of the *Igf2r* cluster has not been tested, loss of EHMT2 has been shown to lead to loss of imprinted silencing of *Slc22a3* [21].

Although it is technically difficult to exclude a role for transcription of the lncRNA versus the RNA product, together these results indicate that imprinted lncRNAs like *Airn* may silence distant imprinted genes like *Slc22a2*, *Slc22a3* and *Pde10a* by recruiting and targeted PRC1, PRC2 and EHMT2 to these genes to deposit repressive chromatin modification and cause silencing. This indicates that *Airn* silences imprinted genes in the *Igf2r* cluster by two different mechanisms: *Airn* transcription silences *Igf2r* by transcription interference that does not require repressive chromatin modifying complexes [7], and the *Airn* RNA product recruits repressive chromatin modifying complexes and targets them to distant, non-overlapped genes like *Slc22a2*, *Slc22a3* and *Pde10a* to cause silencing. Importantly, the mechanism for targeting silencing remains unknown.

**Fig 5:**
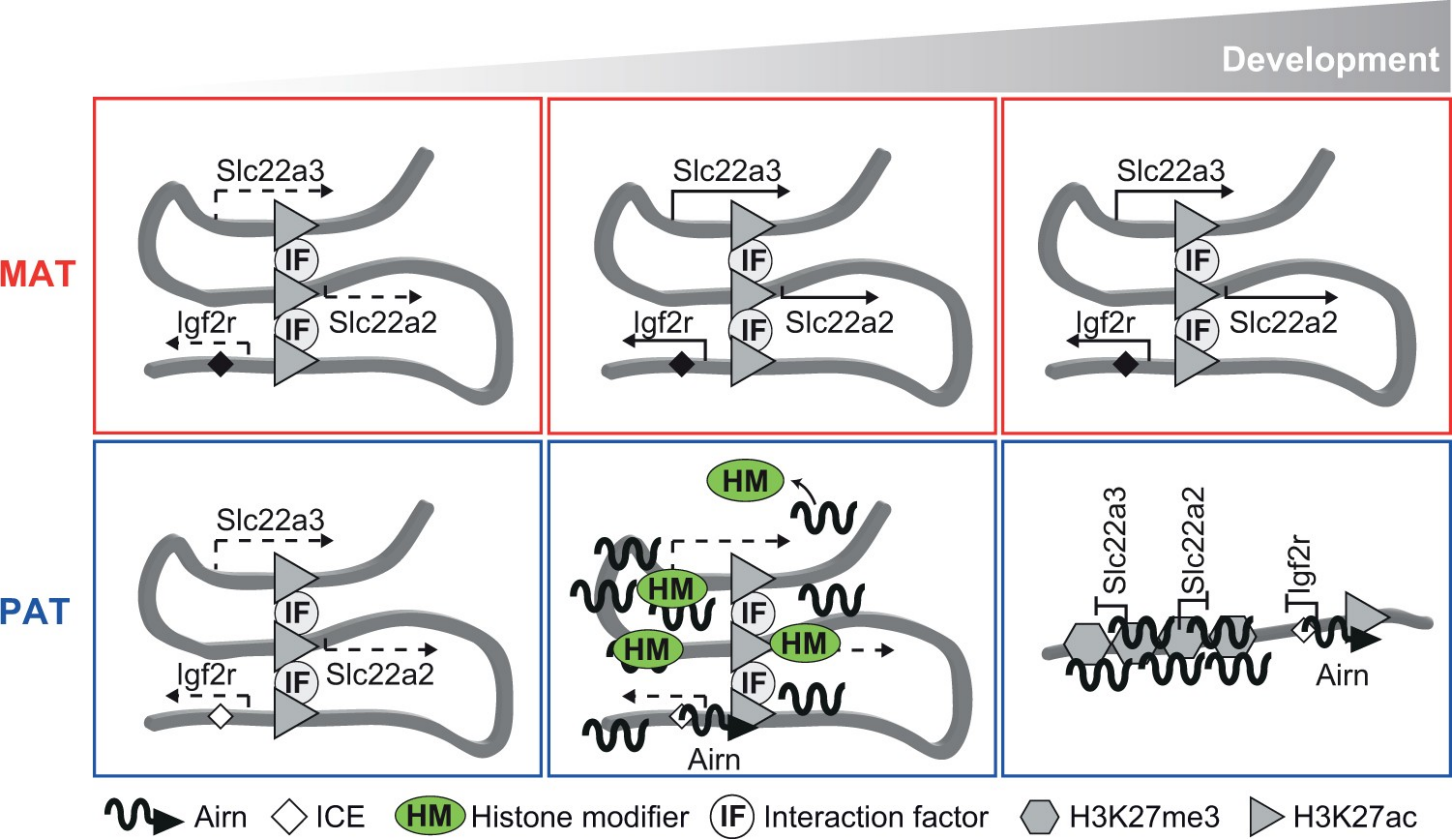
Model: chromosome conformation guides *Airn* lncRNA to target silencing of distant imprinted genes in extra-embryonic tissues. Left: In early development where *Airn* is not expressed, and the genes that it regulates *Igf2r*, *Slc22a2* and *Slc22a3* are expressed at low levels from both alleles (dashed arrows). Both parental alleles have a chromosomal conformation that brings the *Airn* gene body and the promoters of *Slc22a2* and *Slc22a3* in close proximity. These chromosome interactions may be mediated by interaction factors (IF) at regions of open chromatin marked by H3K27ac histone modification (triangles). The imprint control element (ICE) or *Airn* promoter is methylated on the maternal allele (filled diamond) and unmethylated on the paternal allele (unfilled diamond). Middle: As development progresses, methylation on the maternal allele prevents expression of *Airn*, the chromosome conformation is maintained and *Igf2r*, *Slc22a2* and *Slc22a3* are upregulated (solid arrows). On the unmethylated paternal allele, *Airn* (wavy line) starts to be expressed and recruits repressive histone modifiers (HM) and targets them to the promoters of *Slc22a2* and *Slc22a3*, preventing these genes from being upregulated. *Airn* prevents *Igf2r* from being upregulated by transcriptional interference with its promoter [7]. Right: Later in development, on the maternal allele the chromosome conformation is maintained and *Igf2r*, *Slc22a2* and *Slc22a3* are expressed. On the paternal allele, the loss of H3K27ac and the establishment of a compact repressive chromatin domain including H3K27me3, maintains silencing of *Slc22a2* and *Slc22a3*, and leads to the loss of chromosome interactions.

In this study we showed that chromosome interactions between the *Airn* gene body and the *Slc22a3* promoter are enriched on the maternal allele because *Airn* expression represses these interactions on the paternal allele. We showed that these interactions are not required to upregulate *Slc22a3* expression on the maternal allele, indicating that they are not essential promoter-enhancer interactions, but they may serve to place the *Airn* locus and *Slc22a3* promoter in close proximity in the nuclear space. Chromosome interactions can exist in the ground state or be formed during development [41]. Therefore, we propose that in the ground state interactions between the *Airn* locus and the promoter of *Slc22a3* (and likely all other genes silenced by *Airn*, like *Slc22a2*), are present on both alleles (Fig 5 left). During development *Airn* is upregulated on the paternal allele, and these pre-existing interactions allow *Airn* to target gene promoters while recruiting PRC2 and EHMT2 to deposit repressive histone modifications (Fig 5 middle). The establishment of repressive chromatin on the targeted promoters then leads to the loss of chromosome interactions with the *Airn* locus on the paternal allele (Fig 5 right). On the paternal allele the *Airn* RNA and *Slc22a3* promoter are also in close proximity [21]. This may be achieved by the formation of a compacted repressive chromatin domain [19], which may allow *Airn* to continue to find repressed promoters to help maintain silencing despite the loss of the chromosome interactions.

Imprinted genes show tissue-specific expression. In the *Igf2r* and *Kcnq1* cluster, where imprinted silencing is initiated by a lncRNA, genes closer to or overlapped by the lncRNA locus show imprinted expression in multiple tissues, whereas the more distant genes show imprinted expression restricted to extra-embryonic tissues [5,11,17,42]. Our model seeks to explain silencing of these distant, extra-embryonic specific imprinted genes. In the *Igf2r* cluster there is a relatively clear distinction between *Igf2r*, which is overlapped in antisense by *Airn* and silenced by transcriptional interference [7], and other genes in the cluster that are not overlapped and are silenced only in extra-embryonic tissues. However, it has been recently shown that *Slc22a3* also shows imprinted expression in neonatal tongue and adult liver [11], and *Kcnq1ot1* does not overlap the promoter of any of the proximal imprinted genes in the *Kcnq1* cluster that show imprinted expression in multiple tissues [42]. Interestingly, *Kcnq1* itself has been reported to be subject to lncRNA independent imprinted silencing despite *Kcnq1ot1* lying within one of its introns, and to lose imprinted expression during heart development [43]. Therefore, it remains to be tested if the model can explain lncRNA mediated imprinted silencing of non-overlapped genes in all tissues, or if it is restricted to the specific epigenetic environment present in extra-embryonic tissues, known to have unique features such as low levels of DNA methylation [44,45].

Our model has parallels with one proposed with to explain how the lncRNA *Xist* may find its targets during the initiation of X inactivation. *Xist* initially binds at discrete sites throughout the X chromosome, before spreading to cover the whole chromosome [46]. Similar to imprinted lncRNAs like *Airn* and *Kcnq1ot1*, *Xist* recruits and targets repressive histone modifying complexes like PRC1 and PRC2 to chromatin as part of the X inactivation process [47]. These early binding sites correlate with the Hi-C interaction map in undifferentiated ES cells that have 2 active X chromosomes, indicating that pre-existing interactions in the ground state may guide *Xist* to initiate silencing at these sites [46].

Imprinting and X inactivation show allele-specific differences in gene silencing and chromosome interactions within the same cell, making them powerful model systems for uncovering the mechanism of lncRNA mediated silencing. LncRNAs that show biallelic silencing may act in a similar way, but without a picture of chromosome interactions in the ground state detecting distant target genes may be difficult. Future studies should focus on testing the predictions of the model in imprinted and non-imprinted systems.

## Materials and methods

### Ethics statement

Mice were housed and treated according to Austrian law under Laboratory Animal Facility Permit GZ: 311633/2014/9 that was approved by the Office of the Vienna provincial government. Mice were maintained in accordance with the procedures outlined in the Guide for the Care and Use of Laboratory Animals from the NIH, the opinion of the European Group on Ethics in Science, and the European Union (EU) Protocol on the Protection and Welfare of Animals. The Animal Research is covered by Federal Austrian legislation (Law of Animal Experiments 2012 (“TVG-Tierversuchsgesetz”; regulating the “Experimentation on living animals” BGBI. I Nr.114/2012) and the overriding EU and international legislation and codes of conduct. No experimental procedures were performed on the animals so no extra permissions were required.

### Mouse strains

FVB/NJ (FVB) mice were obtained from Charles River. CAST/EiJ (CAST) mice were obtained from the Jackson Laboratory. The FVB.AK-Del(17)T<hp> (*Thp*) mouse (EM:09898) contains an minimum 5.56Mb deletion on chromosome 17 that includes the *Igf2r* imprinted cluster allowing parental allele-specific analysis [27,28]. The FVB.129P2-Airn<tm1Dpb> (*AirnT*) mouse (EM:09895) has a polyadenylation cassette inserted into the *Airn gene*, 3kb downstream from its start site causing it to be truncated and non-functional [5]. The FVB.129P2-Airn-R2D (*R2Δ*) mouse (EM:09897) has a deletion that includes the *Airn* promoter and the imprint control element (ICE) of the *Igf2r* imprinted cluster [18]. Note that the *Thp*, *AirnT* and *R2Δ* mice have been cryopreserved by the EMMA mouse repository (EMMA ID indicated). The *Sod2-flox* mice contain loxP sites flanking the exon 3 of *Sod2* and were made in a 129 ES cell line [30]. The Hprt-Cre mice express Cre during male meiosis and were a kind gift from Simon Hippenmeyer [31]. Note in mouse crosses the maternal allele is always written on the left.

### Derivation of the *RSDel* mouse by targeted meiotic recombination

The *RSDel* mice were created by *Hprt-Cre*-mediated trans-recombination during male meiosis between the remaining loxP site in the *R2Δ* and *Sod2Δ* alleles [18,29–31]. In the first generation *Sod2-flox* mice recovered from frozen embryos were crossed to *Hprt-Cre* mice, while in parallel *Hprt-Cre* was crossed to *R2Δ*, and the offspring of both crosses were genotyped. In the second generation *Hprt-Cre*/*R2Δ* were crossed to *Sod2Δ*, and Hprt-Cre/ *Sod2Δ* was crossed to R2Δ, and the offspring were screened for triple mutant males. In the third generation *Hprt-Cre*/*R2Δ/Sod2Δ* triple mutant males were crossed to FVB females and the offspring screened for the *RSDel* deletion. One *RSDel* male was detected among 72 offspring, and this male was backcrossed to FVB to establish the *RSDel* strain. Note that this strain has been cryopreserved and is stored at IMBA.

### Tissue isolation

Placenta was isolated from E12.5 embryos under a dissection microscope, taking care to remove as much decidua as possible. Visceral yolk sac (VYS) was isolated from E9.5 and E12.5 embryos under a dissection microscope. The whole VYS was used for the chromosome conformation capture (3C experiments), while for RNA isolation and for chromatin immunoprecipitation (ChIP) the VYS endoderm was mechanically separated away from the rest of the VYS after 1–2 hours of DispaseII digestion at 4°C, as previously described [17].

### Chromosome Conformation Capture

Chromosome Conformation Capture (3C) was performed following established protocols with minor modifications [48,49]. To allow the maternal and paternal chromosome to be examined separately at the *Igf2r* imprinted locus we used reciprocal crosses of *Thp* and FVB mice [28]. To determine the influence of *Airn* on interactions we used *Thp* x *AirnT* cross, where *AirnT* mice have a truncated and non-functional *Airn* [5]. We collected visceral yolk sac (VYS) samples from E12.5 embryos, and processed samples for 3C using a protocol adapted from a method designed for cell culture cells with minor modifications [48]. Briefly, single VYS were fixed for 10 minutes in 500μl 2% formaldehyde/PBS at room temperature, before quenching by adding 56μl 2.5M glycine and incubating for 5 minutes at room temperature and then for at least 20 minutes on ice. The liquid was then removed and the samples frozen on dry ice before being stored at -80°C. DNA isolated from the embryonic heads was used to genotype samples by a DNA methylation sensitive Southern blot assay using a EcoRI/MluI digest and a 1013bp probe (chr17:12,741,515–12,742,527; GRCm38/mm10), which detects a 6.3kb (methylated) and 5.0kb (unmethylated) band at the differentially methylated *Igf2r* imprint control element (ICE). Around 28 VYS were then pooled per genotype and thawed on ice and then incubated with 8ml lysis buffer for 15 minutes on ice (1 tab Complete Protease Inhibitor (Roche) per 25ml lysis buffer (10mM Tris-HCl ph8, 10mM NaCl, 0.2% NP-40)). The samples were then dounced in a 15ml glass dounce (Wheaton) with a loose pestle about 30 times, and then 30 times with a tight pestle, before centrifuging for 5 minutes at 2000g at 4°C. The supernatant was then removed leaving a nuclear pellet, which was then resuspended in 1 x EcoRI buffer (Fermentas). Samples were then subject to EcoRI digestion and ligation as previously described [48]. The formaldehyde crosslinks were then reversed by proteinase K treatment (66μg/ml) and heating at 65°C overnight, followed by another 2 hours at 65°C with fresh proteinase K. The samples were then subject to phenol/chloroform extraction, precipitated, then resuspended in TE buffer before being subject to dialysis overnight at 4°C. The 3C material was then again precipitated and the pellet washed x6 with 70% ethanol and x2 with 100% ethanol, before finally being resuspended in 500 μl TE buffer.

We detected 3C interactions by Taqman quantitative PCR following a previously published protocol with minor modifications [49], and by using the standard curve method to analyze qPCR data. Briefly, a primer and Taqman probe were designed near to an EcoRI site on the “bait” EcoRI fragment (e.g. *Slc22a3* promoter) and a “prey” primer was designed near the EcoRI site for fragments in the target region (e.g. *Airn* gene body). All 3C interactions detected in the *Igf2r* imprinted cluster (*Thp* deletion region on chromosome 17) were normalized by dividing by the mean of 2 interactions with the *H19/Igf2* ICE, an independent locus on chromosome 7. To correct for technical and biological variation between experiments the highest interaction level was then set to 1. The primer/probe combinations used are given in S1 Table.

### RNA isolation

Tissue from VYS endoderm or placenta was collected and homogenized in TRI reagent, and total RNA isolated according to the manufacturers protocol (Sigma-Aldrich).

### RT qPCR analysis

Total RNA from E9.5 VYS endoderm and E12.5 placenta collected from *RSDel* x FVB reciprocal crosses was DNase treated using the DNA-free kit (ThermoFisher Scientific), and then converted to cDNA using the RevertAid First Strand cDNA Synthesis Kit (Thermo Fisher Scientific). Reverse transcription quantitative polymerase chain reaction (RT-qPCR) was then conducted using either a Taqman or SYBR Green system using the standard curve method to analysis the data, and normalization to a house keeping gene (cyclophillin A). The RT-qPCR results in Fig 2 are shown relative to the mean of the wildtype controls. The primers and probes used are listed in S2 Table.

### RNA and ChIP-seq

Strand-specific polyA enriched RNA-seq libraries were generated from E12.5 placenta and VYS endoderm from *RSDel* x CAST reciprocal crosses using the TruSeq RNA Sample Prep Kit v2 (Illumina) modified as previously described [50]. For each tissue, a total of 12 libraries were generated: 3x WT and 3x Del (*RSDel* x CAST, maternal deletion cross) and 3x WT and 3x Del (CAST x *RSDel*, paternal deletion cross). Native ChIP was performed using an H3K27me3 antibody (Jenuwein lab antibody 6523, 5th bleed) on E12.5 VYS endoderm from FVB x CAST reciprocal crosses (2x CAST x FVB, 2x FVB x CAST, tissues from multiple litters were pooled) as previously described [51]. ChIP-seq libraries were prepared using the TruSeq ChIP Sample Prep Kit (Illumina). Both, RNA-seq and ChIP-seq libraries were sequenced with a 50bp single end on an Illumina HiSeq 2000/2500. Note, that the H3K27ac ChIP-seq data from VYS endoderm included in this study was described in a previous study [11].

### Allele-specific RNA and ChIP-seq analysis

Allele-specific expression and histone modification enrichment was detected from RNA-seq and ChIP-seq data using the Allelome.PRO program [33]. The SNP annotation file containing 20,601,830 high confidence SNPs between the CAST/EiJ and FVB/NJ strains was extracted from the Sanger database as described previously described [33,52]. For RNA-seq analysis, but not ChIP-seq, SNPs overlapping retroposed genes including pseudogenes were removed (RetroGenes V6 from UCSC genome browser). The *RSDel* mouse was backcrossed to FVB, but the region around the *Igf2r* cluster is likely to have a 129 background as both the *R2Δ* and *Sod2Δ* alleles from which the *RSDel* mouse is derived were made in 129 ES cells [18,30]. Therefore, in our allele-specific RNA-seq analysis we used only CAST/FVB SNPs where the FVB allele was shared with all three sequenced 129 strains (Final SNP number: 16,988,479 SNPs).

We used the following Allelome.PRO parameters for our analysis:

RNA-seq: minread 2 (allelic ratios extracted from debug folder), RefSeq annotation.

H3K27me3 ChIP-seq enrichment: FDR 1%, allelic ratio cutoff 0.7, minread 1, 20Kb sliding windows.

Note: minread = minimum number of reads that must cover a SNP for it to be included in the analysis.

## Supporting information

S1 Table Primer and Taqman probes and combinations for Chromosome Conformation Capture quantitative PCRs (3C-qPCR). (XLSX)

S2 Table Primer and Taqman probes for reverse transcriptase quantitative PCR (RT-qPCR). (XLSX)

S3 Table Chromosome interactions between the *Slc22a3* promoter and the *Airn* gene body are enriched on the maternal allele. Chromosome Conformation Capture (3C) quantitative PCR (qPCR) raw data and analysis for Fig 1A.
(XLSX)

S4 Table Paternal allele chromosome interactions between the *Slc22a3* promoter and the *Airn* gene body are increased following truncation of *Airn*. Chromosome Conformation Capture (3C) quantitative PCR (qPCR) raw data and analysis for Fig 1B.
(XLSX)

S5 Table The *RSDel* maternal deletion does not affect *Slc22a3* expression, whereas the paternal deletion leads to a doubling of *Slc22a3* expression in E9.5 visceral yolk sac (VYS) endoderm. Real time quantitative PCR (RT-qPCR) raw data and analysis for Fig 2D left panel.
(XLSX)

S6 Table The *RSDel* maternal deletion does not affect *Slc22a3* expression, whereas the paternal deletion leads to a doubling of *Slc22a3* expression in E12.5 placenta. Real time quantitative PCR (RT-qPCR) raw data and analysis for Fig 2D right panel.
(XLSX)
