# Correction: The *Airn* lncRNA does not require any DNA elements within its locus to silence distant imprinted genes

**DOI:** 10.1371/journal.pgen.1009151

**Published:** 2020-10-21

**Authors:** Daniel Andergassen, Markus Muckenhuber, Philipp C. Bammer, Tomasz M. Kulinski, Hans-Christian Theussl, Takahiko Shimizu, Josef M. Penninger, Florian M. Pauler, Quanah J. Hudson

In S1 Table, some of the primer sequences have been omitted. The authors have provided a corrected version of S1 Table here.

## Supporting information

S1 Table Primer and Taqman probes and combinations for Chromosome Conformation Capture quantitative PCRs (3C-qPCR). (XLSX)

## References

[1] Andergassen D, Muckenhuber M, Bammer PC, Kulinski TM, Theussl H-C, Shimizu T, et al (2019) The Airn lncRNA does not require any DNA elements within its locus to silence distant imprinted genes. PLoS Genet 15(7): e1008268 10.1371/journal.pgen.1008268 31329595 PMC6675118

